# Equitable access to the COVID-19 vaccines in Africa (ECOVA) findings from a qualitative enquiry in Zimbabwe

**DOI:** 10.3389/frhs.2026.1549192

**Published:** 2026-03-31

**Authors:** Tatenda Dorcas Kujeke, Gracious Madimutsa, Nyasha Masuka

**Affiliations:** 1 School of Computing, University of Portsmouth, Portsmouth, United Kingdom; 2 Department of International Public Health, Liverpool School of Tropical Medicine, Liverpool, United Kingdom; 3 Africa Health Economics and Policy Association, Institute of Tropical Medicine, Antwerp, Belgium

**Keywords:** COVID-19, vaccines, equitable access, vaccine distribution, vaccine access

## Abstract

**Introduction:**

The COVID-19 pandemic exposed significant global disparities in vaccine distribution and access, prompting urgent calls to strengthen national delivery systems. In Zimbabwe and across the African continent, reliance on imports highlighted the need for both robust local distribution mechanisms and enhanced vaccine manufacturing capabilities. Africa's potential to produce vaccines, raw materials, and medical supplies exists, but its realization demands strategic investment and the promotion of public-private partnerships (PPPs). This study aims to analyze Zimbabwe's current COVID-19 vaccine distribution and delivery mechanisms, and explore the potential of PPPs to establish the necessary structures and processes to guarantee prompt access and administration of vaccines, particularly among vulnerable populations.

**Methods:**

We conducted qualitative in-depth interviews with twenty purposively sampled Key Informants, all possessing specialized knowledge of Zimbabwe's Expanded Programme on Immunization (EPI) and the COVID-19 vaccination program. A standardized template, shared across the study consortium, guided the participant selection process.

**Results:**

Our qualitative investigation reveals that equitable vaccine access was hindered by the lack of routine adult vaccination compared to the well-established immunization of children through the EPI program. While utilizing the EPI infrastructure for COVID-19 vaccine rollout was deemed successful and commendable by key informants, it also faced criticism for perceived disparities in rural outreach, favouring urban areas. The stationary vaccination sites posed challenges for vulnerable groups like the elderly and those living with disabilities in rural regions who faced difficulties traveling long distances, underscoring the urgent need for greater efforts to ensure equitable vaccine access across all population segments.

**Discussion:**

Participant perspectives on local vaccine production in Zimbabwe reflect a blend of optimism and apprehension. Many expressed a strong desire for domestic vaccine development, recognizing its potential public health benefits and self-sufficiency. However, a sizeable number doubted Zimbabwe's current capacity to produce vaccines, citing economic challenges. Instead, they suggested forging partnerships with other African nations to collectively establish a regional or continental hub of excellence, sharing costs and reaping mutual benefits. An essential takeaway from this pandemic is the stark revelation of systemic gaps within the healthcare infrastructure. It is crucial to seize this opportunity to fortify the health system, ensuring its resilience in preparation for future pandemics.

## Introduction

Vaccines represent one of the most remarkable achievements in the history of medicine, profoundly impacting public health by preventing and controlling infectious diseases (1). They are intricate and sophisticated medical tools designed to harness the body's natural immune system to protect against infectious diseases (1). The COVID-19 pandemic has underscored existing disparities in the distribution and accessibility of vaccines (2), reigniting discussions on the need to enhance research and development capabilities for vaccines, particularly in Africa. It exposed the urgent need to bolster domestic vaccine production and address prevailing inequities on the continent. Africa possesses the potential to manufacture vaccines (3), vaccine raw materials, diagnostics, and other medical supplies. However, realising this potential requires a strategic realignment of Africa's investment decisions and the promotion of public-private partnerships or product development collaborations. These measures are essential to expanding research and development capacities for vaccines on the continent.

In December 2020, the WHO published the emergency use listing (EUL) for the Comirnaty COVID-19 mRNA vaccine for emergency use making the Pfizer/BioNTech vaccine the first to receive emergency validation from WHO since the onset of the outbreak (4). This EUL opened the door for countries to expedite their own regulatory approval processes to import and administer the vaccine (4). Despite these measures taken by the WHO, these vaccines were beyond the reach of many Sub-Saharan Africa (SSA) countries, especially as a number of them, Zimbabwe included, were not part of the COVID-19 Vaccines Global Access (COVAX) initiative which is an alliance of several established global health institutions that aimed to improve worldwide access to COVID-19 vaccines (5).

Despite calls for the world to learn from past health crises, the pattern of inequitable distribution of life-saving tools was clearly visible in the COVID-19 pandemic. Urges for solidarity among nations were loud in the early stages of the pandemic, with countries committing to the COVAX initiative which sought to first vaccinate 20 percent of all member countries' populations, giving priority to health care providers. From its inception, COVAX invested in the production of a portfolio of vaccines while also laying down the mechanism for equitable distribution. However, the distribution of the COVID-19 vaccine was the antithesis of what had been promised (6). Nationalism replaced the initial spirit of solidarity, high income countries (HICs) avoided buying vaccines through this mechanism and instead signed bilateral agreements with pharmaceutical companies (6, 7). HICs also hoarded the excess vaccines (8) resulting in the exclusion of the populations residing in Africa. This exclusion was particularly impactful as Africa heavily relies on vaccine imports, with less than 1 percent of its vaccine needs being manufactured on the continent. This dependence, particularly for Zimbabwe, invariably caused uncertainties, affecting the ability of the Zimbabwean government to implement mass COVID-19 vaccinations, safeguard the lives and health of the populace, and subsequently rejoin the global economy (9).

The effects of vaccine nationalism are evident in the stark disparities in COVID-19 vaccine doses administered across countries categorized by income levels. As of November 2023, 79.86% of individuals in high-income countries received at least one dose of the vaccine, compared to only 32.82% in low-income countries, highlighting a significant gap against the 70% vaccination target set by the WHO (10, 11).

After observing other regions initiate COVID-19 vaccination campaigns from the sidelines, Zimbabwe, alongside other African countries, eventually received vaccines through the COVAX facility (12). Zimbabwe received nearly 1 million COVID-19 vaccine doses from the global COVAX facility on October 1, 2021 (13). Specifically, the country was supplied with 943,200 doses as part of its efforts to combat the COVID-19 pandemic (13, 14).^1^ The World Bank provided US$6.6 million to support the deployment and management of COVID-19 vaccines in Zimbabwe, to strengthen health system capacity, to improve vaccine tracking and monitoring and to enhance community engagement and vaccine acceptance (15). The Zimbabwean government committed over $100 million to finance COVID-19 vaccine procurement, demonstrating its commitment to ensuring vaccine access (16). Civil society organizations played the role of advocating for greater accountability and equitable access to COVID-19 vaccines in Zimbabwe (17).

Despite receiving aid in the form of financial resources and vaccines, there was limited equitable access to the vaccines. Building capacity for domestic vaccine manufacturing and promoting public-private partnerships for vaccine research and development could help improve Zimbabwe and Africa's pandemic preparedness and response capabilities.

This study had two objectives:.

1. **Primary:** To analyze the current COVID-19 vaccine distribution and delivery mechanisms in Zimbabwe and assess their capacity to guarantee prompt access among vulnerable populations.
2. **Secondary:** To investigate the potential role of **Public-Private Partnerships (PPPs)**, involving civil society and the private sector, in enhancing vaccine equity and delivery.

## Methods

### Participant recruitment

We conducted qualitative semi-structured interviews (SSI) with 20 key informants. Participants were purposively sampled for their specialized knowledge and expertise in Zimbabwe's Expanded Program on Immunisation (EPI) and COVID-19 vaccination programs.

The Africa Health Economics and Policy Association (AfHEA) template guided participant selection to standardize data collection across the study consortium. Participants (≥18 years) were drawn from diverse sectors working in the vaccination space as distributors and policymakers, not as vaccine recipients. These sectors included government, public and private (non-profit and for-profit) organizations, Non-Governmental Organizations (NGOs), civil society, UN agencies, and other health development partners. The in country principal investigator provided a list of potential participants, which two research assistants then used to contact and recruit individuals. Interview appointments were scheduled as either in-person or virtual sessions, according to participant preference and availability.

The sample size of 20 was determined by the depth of knowledge required for the subject matter. Our decision to conclude data collection after 20 interviews was guided by the principle of thematic saturation. To ensure rigor, data analysis was an ongoing and concurrent process that began immediately following the transcription of the initial interviews. The research team engaged in regular, iterative meetings to discuss and map the emerging codes and themes, as well as to identify any conceptual gaps that needed to be explored in subsequent interviews. This continuous approach allowed for the systematic monitoring of theme development. We determined that thematic saturation was achieved when the final set of interviews yielded no new codes or unique thematic insights relevant to the research question, but rather provided further substantiation and depth to the existing thematic framework.

### Data collection

The interviews were conducted from the 28th of March to the 26th of June 2024 by two experienced qualitative researchers and the participants were interviewed in person or virtually on Zoom, Microsoft Teams or over the telephone depending on what medium was most convenient for the participant. This flexible approach ensured the inclusion of all selected participants, regardless of their geographic location within Zimbabwe.

Written informed consent was obtained from all participants prior to their involvement in the study. This consent specifically covered participation in the study and the audio recording of the interviews. For participants who preferred virtual interviews, the written consent form was securely transmitted via email, which they digitally or manually signed and returned to the research team prior to the commencement of the scheduled interview. Nineteen interviews were audio recorded and one participant opted out of being recorded. The researcher took detailed notes during the interview with the participant who opted out of being recorded. The notes were then transcribed after the interview. All audio files were transcribed verbatim and stored on a secure drive for analysis.

The interviews were mostly conducted in English although some participants switched to their vernacular language, Shona, to emphasise points they felt were important to note. Both the researchers understand both languages and the Shona was translated and transcribed in the 20 transcripts.

### Setting

Out of the 20 participants, 17 were from Harare and three were from three different provinces outside Harare. Harare is the capital city of Zimbabwe and is also the centre for government ministries and administrative functions.

### Ethical approval

Ethical approval was granted by Medical Research Council of Zimbabwe (MRCZ) (approval number MRCZ/A/3151) on the 26th of March 2024.

### Data analysis

All files were anonymised and saved on password-protected computers. All data were then transferred to a qualitative data analysis software package, NVivo 12 (QSR, Melbourne, Australia). Braun and Clarke's thematic analysis was employed to investigate the experiences and perceptions of the participants during the pandemic. Codes were developed based on emerging themes from the data and the topic guide, and the interviews were organised based on the codes, and explored to identify emerging themes from the data.

## Results

### Socio demographic profiles of key informants

15 (75%) of the participants had attained Master's degrees and the minimum qualification was a bachelor's degree. 10 (50%) had been in management or leadership positions for 5 years or more. The average age of the interviewed participants was 47.5 years old. Their comprehension of the questions was not compromised in any way as they were able to respond. Most of the interviews were conducted online, only 8 (40%) were conducted in person. While conducting most interviews virtually may have limited non-verbal communication, the non-sensitive nature of the questions minimized the potential for social desirability bias. There was a lower representation of women (4 out of 20) in the sample. However, this demographic imbalance did not compromise the quality of the data, as key informants were selected from a pool of high-level professionals in a field that is currently male-dominated Table 1.

**Table 1:** Socio-demographic profiles of key informants.

Characteristic	*n*
Age	
Mean Age	47.5
Age Range	34–63
Highest Level of Education	
Bachelor's Degree	4 (20%)
Master's Degree	15 (75%)
Doctor of Philosophy	1 (5%)
Years in management or leadership position	
0–2 years	5 (25%)
3–4 years	5 (25%)
5–+	10 (50%)
Gender	
Female	4 (20%)
Male	16 (80%)
Type of Organisation	
Government	8
Private Organisation	5
Non Governmental Organisation	2
Parastatal	2
Civic Society Organisation	2
Private Not for Profit	1
Mode of Interview	
In person	8 (40%)
Virtual (MS Teams/Zoom)	11 (55%)
Telephone	1 (5%)

### Vaccine access

One of the challenges that the country faced when the vaccine was rolled out was that the adult population was not routinely vaccinated compared to the young children who are reached through the Expanded Programme on Immunisation (EPI) program with routine vaccination campaigns. Zimbabwe addressed this obstacle by leveraging the established EPI program to distribute the vaccine, ensuring widespread access. This strategic decision was widely praised by the majority of participants.

“We don't routinely vaccinate adults … so they … they tried to use their EPI [Expanded Program on Immunisation] which is really well done for childhood vaccines but has never had to deal with vaccines for adults. They used their existing structures and I think they did pretty well actually. Just using existing structures to try and get access to vaccines” **ECOVA_KII_011, Female**

“…the thing with our vaccine distribution, it's not coming in as a new thing totally because prior to the Covid-19 pandemic, there were other vaccines that were being distributed utilising the ZEPI [Zimbabwe Expanded Program for Immunization]  … so, when the Covid-19 vaccination program came into play, it just rode on the already existing program” **ECOVA_KII_017, Male**

Although utilising the established EPI framework was a commendable strategy, challenges emerged regarding vaccine accessibility due to insufficient resources. These included a shortage of vehicles for vaccine transportation, a lack of healthcare personnel for distribution in remote areas, absence of allowances to incentivize and compensate healthcare workers for their work in risky conditions, inadequate fuel supplies, and a scarcity of staff to administer the vaccines. Staff shortages, exacerbated by high turnover rates, led to burnout, COVID-19 infections, and migration of healthcare workers seeking better opportunities outside Zimbabwe.

Some participants described vaccine access as suboptimal in rural, hard-to-reach areas, peri-urban regions, and newly developed formal and informal residential areas lacking health facilities. They expressed concerns that centralising vaccination centres restricted access primarily to urban residents and those near designated vaccination centres. Additionally, the absence of mobile vaccination units most likely due to inadequate funding, further impeded accessibility, particularly affecting elderly individuals unable to travel long distances and people with disabilities who are immobile.

“I know Covid vaccination was somehow centralised … I remember the first centre was Wilkins, then BRIDH [Beatrice Road Infectious Diseases Hospital], then central hospitals came in. I don't remember having people in the rural areas having mobile vaccination teams. Maybe I didn't hear about it … from where I come from in[Village name withheld], my family, I don't remember anyone getting vaccinated … they had to travel to Bindura I think or Mt Darwin district hospital” **ECOVA_KII_015, Male**

“… in terms of resources, when our vaccinations are being conducted, as a country, we deal with static vaccinations where clients would need to visit health institutions to get the vaccines. We also talk about mobile teams which will be roving around the cities or communities so that whichever groups of people they encounter, they then provide them with vaccination. So usually the biggest numbers come from the mobile teams, the static teams do not usually contribute to significant numbers compared to the mobile teams, and these have to be moving around and the fact that they are moving around means that they require more costs, they require transport, fuel and there is also need for those daily subsistence allowances so that they come in as a motivational factor for pushing of numbers. So the latter is quite costly regardless of the fact that it pushes numbers, it's quite costly, it comes with a price. Our country is a low socioeconomic one that means to a certain extent our country is incapacitated, resources are never enough. So that's one of the things we encounter. We are not well resourced enough to be pushing numbers and mobilising teams so that they facilitate the vaccination programs.” **ECOVA_KII_017, Male**

### Vaccine hesitancy

A common thread throughout the interviews was the prevalence of vaccine hesitancy among eligible recipients in Zimbabwe. Participants attributed this hesitancy to various factors. Primarily, the ever-evolving nature of COVID-19 led to frequent guideline revisions as scientists unearthed new insights about the virus and potential treatment protocols for the infected. While the dynamic nature of these guidelines was seen as progressive from a scientific perspective, the general populace encountered a stream of constantly changing and often conflicting information. This inconsistency heightened suspicions and further fuelled their reluctance to receive the vaccine.

“…we were even under-resourced in terms of information because the accurate information about Covid-19 vaccine and Covid-19 in general kept changing. This week we would have new information at first, we thought it was airborne then it was now droplet infection. It kept changing so, that thing that we did not have authentic or profound information about Covid-19 vaccination, so it kept us on the tail end of everything.” **ECOVA_KII_017, Male,**

Vaccine hesitancy was not confined to the general population; it also extended to healthcare workers on the frontline of the pandemic. Even after witnessing the Minister of Health, who was also the Vice President at the time, receive the vaccine on live television to emphasise its safety, some healthcare workers remained hesitant. Many of them only opted for vaccination when mandates were issued requiring all healthcare workers to be vaccinated, often accompanied by a risk allowance as an incentive.

“I’ll tell you that we were the first people to be vaccinated at [the hospital]with the Sinopharm vaccine from China. So we had to rope in the Vice President of the country, the country leadership was very supportive. So the Vice President came, he was then the Minister of Health, he was vaccinated first, the Deputy Minister was also vaccinated then the Directors from the Ministry were also vaccinated. So that also helped. But for your information, healthcare workers, even the nurses that were assisting Covid-19 cases at Wilkins were not vaccinated on that same day … and some of our colleagues who were doing health promotion … they did not get vaccinated, they wanted to see how things would occur. Even journalists were there, they didn't get vaccinated. But doctors were forthcoming with the vaccination than nurses” **ECOVA_KII_018, Male,**

“I think vaccine mandates really did help particularly in the civil service particularly with health care providers because we saw a surge in coverage among health care workers when the mandates were also now there. I think government was also clear that if you become Covid-19 positive yet you are not vaccinated, the risk allowance might be limited or low because you needed to make sure that you used every means available to protect yourself and if you get the Covid-19 then it would make sense for government to support you. So those mandates also helped.” **ECOVA_KII_016, Male,**

The broader population followed suit, primarily getting vaccinated when mandates were imposed, especially in workplaces for formally employed individuals. Others were motivated to get vaccinated after witnessing acquaintances, community members, or family fall ill with the virus or after experiencing the loss of loved ones in their communities or families.

“…so those that have relatives that succumbed to Covid-19, those that noticed people getting sick, they were now afraid that this thing actually kills so they decided to get the injection. There are others who remained adamant.” **ECOVA_KII_013, Female,**

Participants also highlighted another factor contributing to vaccine hesitancy: the origin of the vaccines available in Zimbabwe from the People's Republic of China. The general population often associates products sourced from China as “cheap” or lacking in efficacy, based on previous encounters with low-quality and non-durable goods from the country. This perception extended to the vaccines, with some believing they would be ineffective and harbouring suspicions that China aimed to reduce Zimbabwe's population by causing infertility through the vaccines.

“People were anxious about the vaccines, they were Chinese vaccines and there is still an attitude about the quality. Quality is not something people associate with China so there was a lot of mistrust on the source of the vaccines. There was fear of what would happen in pregnancy and we are a fertility oriented culture and so I think at first there was misunderstanding in pregnancy. There was fear from misinformation about what would happen to you. Many people thought it's the West trying to kill you and in a few years you will be dead or in five years you would be dead.” **ECOVA_KII_011, Female**

“…there is this information gap, this was a challenge. You know when there is information all over, and a lot of misconceptions, a lot of lies, negative energy against vaccination was running rampant like wildfire … so the health ministry was always playing catch up. One day, a message would just pop up on the internet claiming that this vaccination would lead to sterilisation … or you will die within two years” **ECOVA_KII_017, Male**

Religious communities, notably members of the Apostolic sect, emerged as a significant challenge in vaccine acceptance efforts. Convincing these communities to vaccinate proved particularly daunting due to deeply held beliefs that led to widespread vaccine refusal within this group. Engaging with and addressing the concerns of these religious communities were crucial steps in overcoming this obstacle to achieving broader vaccination coverage.

“…those who are extremely religious like the Apostolic sects from the ‘Madzibaba’ or whatever clans who would say ‘we were born without any medical interventions so we remain strict as far as vaccination is concerned, we will not get it’.” **ECOVA_KII_017, Male**

A participant underscored the impact of insufficient vaccine supplies on public willingness to vaccinate or attend follow-up appointments. It was noted that a particular vaccine ran out after individuals had already received the first dose. Consequently, they had to restart the vaccination process with an alternative vaccine. This unexpected change often elicited negative reactions from the affected individuals.

### Vaccine manufacturing

Participant views on vaccine manufacturing in Zimbabwe reveal a mix of optimism and concern. A good number of the participants expressed a strong desire for vaccines to be developed locally, recognizing the potential benefits this could bring to public health and self-sufficiency. However, a significant number of participants felt that Zimbabwe currently lacks the necessary capacity to produce vaccines, primarily due to the country's economic challenges.

“… there is a drive to manufacture vaccines, but again the macroeconomic conditions then defeat the whole purpose. There are two factors that come into play. The first one being the uncompetitive nature our inputs vis-a-vis other countries which makes the cost of production very high in Zimbabwe … and number two, the technology and the inputs, the quality of inputs that are required for one to be able to manufacture vaccines. I think we are not there yet.” ECOVA_**KII_003, Male**

“It's not hopeless, but it's good to think about it [vaccine manufacturing], but at the same time have the conducive environment for that to happen. You can think about or you can desire to have manufacturing, good manufacturing practice, but if the environment is not conducive or it's not enabling you may continue thinking about it, but it may not gather any ground in terms of progress because of the issues that are beyond the control, like I said, power, cost of labour in Zimbabwe is generally high.” ECOVA_**KII_003, Male**

Capacity building was cited as one of the major requisites for the country to initiate vaccine manufacturing. Participants expressed a strong belief that with the right investments in STEM [Science, Technology, Engineering and Mathematics] education, training, infrastructure, and technology, the country could successfully embark on this endeavour.

“I think in the long run, like other countries are starting to do, we need to start thinking about building capacity, local capacity for vaccine manufacturing. We can start somewhere, it may not be COVID-19, but we have to start thinking about building capacity for vaccine manufacturing locally.” ***ECOVA_* KII_001, Male**

“I think there is need for us to start thinking of developing our own manufacturing sites to create or to manufacture these vaccines so that we don't waste much of our resources importing these products from outside Zimbabwe and we also create employment if we start manufacturing in Zimbabwe and this will create employment and lessen our burden in terms of foreign currency etc.” **ECOVA_KII_004, Male**

“STEM [Science, Technology, Engineering and Mathematics] investment is so critical … I would say for Africa, yes, dream about manufacturing but the real work is the hard political work, invest in STEM, build the middle class, build the economy, build the market … You have to build STEM and look at how countries develop. Study that then do that. That's where China is an interesting model because they looked at the United States and said ‘you know what, the way to become a super power is to invest in STEM, in science’” **ECOVA_KII_011, Female**

Even though few participants were aware of any tangible efforts in starting vaccine manufacturing, some highlighted some tangible moves that were being done by some organisations in trying to initiate vaccine manufacturing. There was a prevailing sentiment that the government is appreciative of and supportive toward these efforts.

“So MCAZ [Medicines Control Authority of Zimbabwe] have approached us sometime and they want to set up some manufacturing plants for these vaccines … and I think last week we had a meeting with one of the people who want to develop their own vaccines here in Zimbabwe and I think there is support from the government in terms of resources to use in setting up these facilities so that we can be able to produce our own vaccines.” ECOVA_**KII_004, Male**

Participants expressed limited awareness of companies specifically involved in vaccine manufacturing. While they were familiar with the production of other pharmaceutical drugs, they noted a lack of knowledge regarding companies that focus on vaccine development.

“I'm not aware of any entity that wants to go into vaccine manufacturing, but I know that there is quite some activity just around manufacturing pharmaceuticals. We have two new companies that are doing manufacturing, but they're doing  … what in our industry may call the easy sort of manufacturing, you know, solid dosage forms, your tablets, suppositories. When it comes to vaccines, these are sterile products. I think the closest thing that used to happen to vaccines in terms of manufacturing were the … the vacillators that you know your normal saline rearing and lactate those used to be manufactured by a subdivision of Caps which was called auto sterile.” ECOVA_**KII_009, Male**

### Views on public-private partnerships (PPP)

Participants expressed diverse views regarding public-private partnerships (PPPs) in Zimbabwe. There was a general perception that PPPs were advantageous for the health sector during the COVID-19 pandemic, as they facilitated the sharing of the pandemic's burden between the government and private entities. Many participants noted that the upper and middle classes were most affected by COVID-19, which necessitated the involvement of the private sector, particularly hospitals, in pandemic management. This was due to the preference of wealthier individuals for private healthcare services over public hospitals. While COVID-19 vaccinations were primarily available at public hospitals free of charge, the inclusion of private hospitals contributed to increased vaccination coverage. One participant highlighted a specific PPP between the government and a private hospital, which helped promote equity in vaccine distribution.

“… epidemiology was really characterised by the surprising finding or the surprising thing that quite a few of the affluent middle-class kind of well-to-do or have means people were the ones that had a more visible COVID problem in Zimbabwe and this is a segment of the market that does not ordinarily go to a public hospital for service, or public clinic right? Or a public clinic, they go to private doctors. They go to private pharmacies and if they are not medical insurers, they can afford out of pocket expenditure. The government realised that it's a powerful constituency and it also relieves the burden of the national fiscus. So that's how we came up with an arrangement where we had the Arundel of hospitals stepping in, it was a public-private partnership thing.” **ECOVA_KII_005, Male**

“I think it [PPP] includes … it expands coverage in terms of vaccination. So like I said, the CIMAS and the Ministry of Health partnership, it actually increased coverage in terms of vaccination program because they vaccinated quite a number of their clients and it also helps to reduce congestion in the public sector because some of the people will be handled by their private player and it also helps in terms of managing because we know sometimes the public sector does not have adequate resources in terms of human beings or human resources**.” ECOVA_KII_004, Male**

PPPs were not only beneficial in ensuring coverage of services to the affluent members of the community. They were also very useful in complementing the limited resources of the government to ensure that vaccine delivery was smoother. For example, the private sector came in to assist with storage facilities to ensure the cold chain was maintained. Other private companies came forward and offered to pay for their specific vaccines and these vaccines were procured through the government. This was a win-win situation where the government benefited by getting financial assistance to curb the COVID-19 problems and the private sector also benefited by supporting an initiative that ensured that they didn't lose their human resources through the demise of their workers as a result of COVID-19. By partnering with their resources, these partnerships helped ease the financial burden on the government and this allowed for the vaccination programme to be implemented more efficiently, leading to a healthier workforce in the private sector.

“For their boosters, those ones, the Econet ones. Yes, so there was a partnership where Econet assisted in provision of the energy to keep the vaccines cool.” **ECOVA_KII_001, Male**

“CIMAS just came into my mind, but there are a number (of private organisations) that also put in their money there because they want, companies were scared (feared to lose their employees to death as a result of COVID-19). They knew if people drop dead, you have no company to talk about. People needed to be protected. People needed to be vaccinated. They couldn't go and buy on their own. I mean, if you have a company of a thousand people, you're not a contender or you could not go and purchase these vaccines from wherever they were being purchased. Those selling them needed the big ones, you know, and you remember the United States, UK, they all had their vaccines even hoarded. Small companies from countries like Zimbabwe could not compete. So this collaboration with the government became so handy and very useful and we did do that.” **ECOVA_KII_002, Female**

While PPPs were initially viewed as highly beneficial for both government and private sectors, participants expressed concerns about the accountability of the private sector in delivering services to established standards. The government's management of vaccine distribution ensures that it operates in the best possible manner, as the responsibility for vaccine delivery lies with them. Involving the private sector introduces the risk that some protocols may not be followed as intended, since the methods employed by private companies can differ significantly from those mandated by the government. This divergence raises challenges in effectively monitoring the quality of services provided by the private sector. However, during the pandemic, the Ministry of Health and Child Care (MOHCC) and local governments provided training to private healthcare providers interested in offering vaccination services to ensure adherence to standardised operating procedures for vaccine administration and reporting statistics on vaccinations and adverse events.

“The quality of the services that they [private providers] are providing … are they keeping the vaccines in the appropriate storage conditions … temperatures, are they using the appropriate syringes and needles to vaccinate? Are they vaccinating at the appropriate sight? Yes, are they providing the after—vaccination support and monitoring of the patients? So, all that is to be monitored and supervised that the quality of vaccinations is maintained. You know, public confidence in vaccinations can easily be ruined by one or two events.” **ECOVA_KII_001, Male**

“It was more of a public private partnership where we then trained … I was actually in the forefront of training all the private doctors and private practitioners … we would conduct daily trainings from 8 to 10 am giving the theoretical work then the practical work. We did this at Beatrice Road Infectious Disease hospital [BRIDH], on actual patients so they [private providers] understood what was going on. The doctors would then order their vaccines direct from BRIDH, from our provincial so that they would administer. We had a reporting system, because we conducted a training on how to report on summary sheets where they would record usage, wastage, monitoring of cold chain, expiry dates. So, we created a WhatsApp group where they would then post updates of whatever workload they had for the day. If they had not done any for the day they would record zero for that day. It was quite formalised” **ECOVA_KII_013, Female**

Another concern that was raised by participants was that adverse events may not be handled well by the private sector and this may potentially tarnish the whole vaccination programme. While participants did not completely dismiss PPPs, they emphasised the need for government oversight to ensure the programme success.

“So if there are errors there on that side, people don't say it's the private sector, they say the vaccine and you can kill the whole vaccination program. So in this partnership, in this collaboration, there will also be a need for that kind of oversight to ensure quality and safety of vaccines is maintained all the time.” **ECOVA_KII_001, Male**

Even though it was evident from the interviews that PPPs were existent in the country, one participant had a different view. He highlighted that even though people talk about PPPs a lot, there is no legal framework for them to work because they are not sustained by the law.

“First and foremost, there is no framework for public health private-public partnerships. People talk about it a lot, but there is no legal framework for it in Zimbabwe. So it's difficult to then talk about a partnership that cannot be sustained by law, because whatever we do as a ministry should be backed by either a digital instrument or an act of parliament that enables us to do that.” **ECOVA_KII_003, Male**

### Equity in vaccine distribution

Participants presented diverse definitions of vulnerable populations, with viewpoints shaped by their respective areas of expertise. They identified vulnerable groups as individuals on the pandemic's front lines in terms of service delivery, including healthcare workers of varied specialties, immigration officers at all entry points, individuals with disabilities, those with comorbidities, prison populations, refugees, children, pregnant women, and residents of rural and hard-to-reach areas.

“those living with disabilities, those living in closed institutions such as the prisons because in terms of mode of transmission and their environments, also those living in crowded houses because of WASH issues, they are vulnerable. Even working in health settings” **ECOVA_KII_013, Female**

“so, I think those who are exposed to high-risk areas particularly health care areas, are high risk because they come into contact with a high number of people. I think we lost quite a high number of health care workers to Covid as a result of the services they provide so that's a high-risk population. And then the people who are working at the ports of entry, the immigration guys, I think they also come into contact with people coming from outside Zimbabwe and if Covid-19 or any other related virus is not detected, those guys are also at high risk of contracting the disease. And then we also look at people who are elderly because of their diminished immune system they become highly vulnerable to Covid-19 or some related viruses and the same goes for our constituency, people living with HIV I think they are also vulnerable particularly in such situations where we are looking at a highly transmittible virus like Covid-19 or a related one, they also gather in health facilities and those are high risk places which also place them at an increased risk of contracting the virus” **ECOVA_KII_012, Male**

Opinions on equity varied among participants. While some believed that the program effectively promoted equity by striving to provide vaccine access to diverse populations during the 2020–2021 vaccination campaign, others expressed concerns about the lack of equity. Criticisms centred around the perceived disparity in reaching rural areas, with distribution appearing to favour urban residents over their rural counterparts. This highlighted a consensus that more efforts were needed to address equity issues, particularly in ensuring equal access to vaccines for all segments of the population.

“… so, I think equity issues were also a cause for concern because I think you would agree that the urbanites [those in urban areas] were also easily accessing those vaccines compared to those in the rural or hard to reach areas, so in terms of equity I think it's something we can't conclusively say there was equity. Of course, the government said they reached all the places, but we can't really compare the equity in terms of those in urban areas and rural areas. And also, we can't also talk of equity particularly in terms of accessing vaccines for people in need. I think towards the end there was high demand for vaccination among the population but unfortunately the demand was not met from the supply side and for us it was quite an issue.” **ECOVA_KII_012, Male**

“I would say the ministry tried because I remember the first batch that received vaccination were the healthcare workers, they were given first preference because they were the ones at the forefront of the pandemic, so yes there was equity. Because if it was equality we would just open the gate to everyone and say whoever is first in line will get a jab. So, I guess there was equity because we started with health care workers who were vulnerable, then we went onto the above 65s because those ones have non communicable diseases etc. then we later moved onto the rest of the population. So, there was equity, we did try.” **ECOVA_KII_017, Male**

Looking at men and women, there were varying views regarding equity in their access to vaccines. Some participants indicated that women were generally disadvantaged when it came to access to vaccines because of the various intersecting vulnerabilities that they face as women. Firstly, Zimbabwe is patriarchal, and most women are left with the responsibility of taking care of the children at home while men go to work. Most women must seek permission to leave the home or to even take the vaccines, thereby placing them at the mercy of the patriarchal figure with authority over their lives. Equity was even more difficult for the rural woman who was faced with the challenges of dealing with lack and harsher realities of patriarchal dominance that impeded their abilities to actively seek vaccination services or to participate in whatever vaccination programme available to them.

“Mmmm It's hard to say, but I mean for our society, I think I can almost generally assume that men in general have more access to everything than women, although we've generally improved over the years. But I think in general, many more access for a lot of reasons, economic reasons, social reasons, the ability to then move to go and get these vaccinations, access to information. Yeah so especially when you look at it in the lower income settings, perhaps it's just the men who have some kind of income, they can travel to the vaccination centre or they're the one that goes to work and maybe at their work site. I think it would have been planned that they get a vaccine. I know there's some workplaces where vaccinations happened. So I think given the general setup of our society, I'll tell you it would have been more biased to men. I don't have data. I will just use my assumptions and my opinions to come to that position.” **ECOVA_KII_009, Male**

“Obviously, women are mostly disadvantaged because of the nature of their work, they are usually preferred to be in their home and obviously, there is a perception that women should not get the vaccine because they will be at home. And there are also those of gender dynamics in terms of access to vaccine because definitely women were almost highly disadvantaged in terms of access to vaccine.” **ECOVA_KII_007, Male**

Despite noting that equity was compromised between genders, a good number of the participants were of the opinion that there was equity and both men and women had the same opportunities to get vaccinated for Covid-19.

“Yes, there was equity in terms of vaccine distribution and women had the same opportunities.” **ECOVA_KII_008, Female**

For some participants, the exclusion of women stemmed from fears regarding potential risks to their pregnancies, and they argued that there was no evidence that it was medically safe for the women to be vaccinated whilst pregnant.

…at one point, I think we were actually seeing pregnant women excluded and part of it was just the fear that it may have an impact on their pregnancies and it wasn't proven but they were excluded. So, and there was no medical backup or proof that they should be excluded but they were excluded from vaccination because of gender. So I don't think we actually managed gender equity. **ECOVA_KII_010, Male**

The participant further supported his claim by arguing that women have better health seeking behaviour than men. Another participant also insisted that there was vaccine equity between men and women because they had the same opportunities.

“Ah … I think the other, maybe potential issues that men in general don't really have good health seeking behaviour compared to women. Women tend to be more … they take care of themselves more than what men do. So men would just walk around, whether they are vaccinated or not, they really don't care. So maybe that could have impacted … . I'm not sure about the male-female ratios in terms of our vaccination statistics, but yeah.” **ECOVA_KII_010, Male**

## Discussion

In this study, our primary objective was to analyse the current vaccine distribution and delivery mechanisms in Zimbabwe, examining the necessary structures and processes to guarantee prompt access to and administration of COVID-19 vaccines, particularly among vulnerable populations. We also sought to investigate the potential role of PPPs and product development collaborations in advancing vaccine research, development, and dissemination within the country. Our findings revealed a myriad of strategies and recommendations put forth by participants to bolster equitable vaccine access. Notably, participants underscored the pivotal role of PPPs in mobilising resources that would otherwise be inaccessible, especially in the realms of vaccine research, development, and distribution.

Constructing sustainable vaccine manufacturing and distribution capabilities in developing nations holds promise for mitigating global supply and research disparities (18). However, the intricacies of vaccine production and distribution demand stringent quality assurance measures and rigorous regulatory oversight (7, 18, 19). This undertaking is financially intensive (20), posing a significant challenge for developing countries already grappling with diverse economic priorities. An alternative approach to domestic vaccine production could involve collaboration among African nations (19), either regionally or continentally, pooling both human and financial resources to establish a centralized excellence hub. Each member country would contribute financially to this centre, streamlining the learning process and alleviating the burden on individual nations to set up manufacturing facilities and navigate post-saturation vaccine markets. Research indicates that establishing a vaccine production facility could necessitate investments ranging from US$60 million to US$130 million (20). Given Zimbabwe's economic context, such a substantial cost could present a formidable obstacle for the country (9).

According to the WHO, ten African manufacturers are actively engaged in vaccine production, spanning five countries (Egypt, Morocco, Senegal, South Africa, and Tunisia) (8). Should Zimbabwe venture into vaccine manufacturing, it might not hold a competitive edge due to various obstacles such as the high cost of building manufacturing plants (21), the high cost of conducting business in Zimbabwe, coupled with the complexities of company registration (22) and bureaucratic hurdles (23), which act as a deterrent for potential investors. Moreover, persistent power outages (24) necessitating costly generator usage would inflate operational expenses, particularly in maintaining the essential cold chain for vaccine viability, ultimately impacting company profitability. Collectively, these challenges indicate that local vaccine manufacturing in Zimbabwe remains a distant prospect. Collaborating with other African nations, and leveraging their comparative advantages, may represent a more viable path forward.

The lack of domestic vaccine manufacturing capabilities means Zimbabwe is heavily reliant on vaccine imports from establishments such as the Gavi Alliance and COVAX. This has implications for the equitable access to vaccines, as it places the country outside of the control of supply chain challenges that emanate from relying on other countries or organisations to provide vaccine needs.

Additionally, since the adult population in Zimbabwe is not routinely vaccinated, the COVID-19 vaccines were distributed using the existing Expanded Programme on Immunization (EPI) system, which normally targets and reaches the vaccine needs of children. While leveraging the EPI system yielded notable results, many adults—particularly those residing in rural, hard-to-reach areas, the elderly, and those living with disabilities—were not reached in the vaccination drives. This is because the vaccine centres were predominantly stationed at static health facilities which these groups could either not travel to due to mobility challenges or could not afford to access.

These findings are consistent with a previous study by Cao et al. (25), which highlighted that in Zimbabwe, over 70% of the population had to travel over 60 min to the nearest vaccination centre, with some instances showing almost no vaccination sites available. This represents a significant impediment to vaccination efforts, considering 67% of the population resides in rural areas (26).

Looking to the future, it is important to implement strategies such as mobile vaccination centres in addition to the existing distribution mechanisms to address inequities in vaccine access. This is where Public-Private Partnerships (PPPs) can play a critical role in ensuring this equitable access.

PPPs have the power to transform healthcare through interventions in research (collaboration to develop new technologies and solutions), resources (financing for infrastructure and healthcare capacity build-up) and remittance (development of execution capabilities, skills and knowledge for healthcare delivery) (27). Throughout the pandemic, PPPs played a key role in expanding vaccine access to a broader demographic, especially benefiting individuals in the middle and upper classes through their medical insurers and private healthcare facilities in Zimbabwe. A notable example of a successful partnership model in vaccine development is India, where the government and the private sector joined forces to bring safe, effective, and affordable vaccines to the market in a relatively short time (27). The government invested approximately US$110 million which supported clinical trial sites for several vaccine candidates (27). Closer to home, is another promising example, the Biovac Institute which is a PPP between the South African government and the Biovac Consortium. This institute was set up in 2003 to manufacture and distribute affordable, high-quality vaccines for Africa. It now supplies over 15 million doses of vaccines each year to South Africa's provinces, focusing on tuberculosis, measles, pneumonia, and hepatitis B and has successfully reduced costs and made vaccines accessible to the underserved African market (3).

To further bolster PPP initiatives, it is vital to formalise agreements legally to prevent private entities from profiteering in future health crises. Past instances have revealed situations where populations were subjected to excessive fees for vaccines that should have been freely accessible, underscoring the necessity for robust legal frameworks.

This study observed that private sector involvement can sometimes compromise service quality. Similarly, existing research underscores how private sector participation in public services can result in protocol deviations, as private entities may not be bound by the same regulations as government bodies. This divergence can complicate the monitoring of service quality, given that the government ultimately bears responsibility for public health outcomes, including vaccine distribution (28). Therefore, it is crucial for the government to provide comprehensive training on expected standard operating procedures and clearly communicate that any deviations will lead to penalties. This is particularly important as the private sector is primarily profit-driven and may prioritise financial interests over public welfare if it affects their profits. Furthermore, Civil Society Organizations (CSOs) can play a pivotal oversight role, acting as accountability agents that represent the needs of the wider community and help ensure the private sector operates ethically during public health emergencies.

### Next steps

Looking ahead, it is crucial to draw lessons from the COVID-19 pandemic and proactively prepare for potential future pandemics by strengthening the healthcare system to enhance its resilience. This can be accomplished not only by training healthcare workers but also by taking measures to retain them within the country. Ensuring competitive salaries and providing non-financial incentives can help retain healthcare professionals, reducing turnover rates. Retaining these workers in their roles can alleviate the strain caused by shortages and prevent burnout, a significant issue during the pandemic when healthcare workers resigned in search of greener pastures or had to isolate due to COVID-19 infections.

Moreover, it is essential to equip the Ministry of Health and Child Care (MOHCC) with the capacity to procure or engage in public-private partnerships for local manufacturing of personal protective equipment (PPE). This ensures that during future outbreaks, when global supply chains are disrupted or nationalism hinders access, healthcare workers have the necessary PPE.

While Zimbabwe boasts a functional surveillance system that played a pivotal role in managing the COVID-19 pandemic, there is room for improvement through investments in technology. Enhancements such as internet connectivity, backup power supplies, and the provision of devices like tablets, mobile phones, or laptops can facilitate the swift transmission of vital data across all healthcare facilities. The country has previously benefited from collaborations with local telecommunication companies, leveraging their platforms to disseminate verified information on disease outbreaks and enabling the MOHCC to transmit COVID-19 data from health facilities in the communities to the Public Health Emergency Operations Centre (PHEOC) facilitating rapid real-time decision-making about resource allocation.

This presents an opportune moment for the MOHCC to acquire suitable refrigerated vehicles for vaccine transportation, ensuring the maintenance of the cold chain integrity to preserve vaccine efficacy. Additionally, procuring vehicles to transport healthcare workers to remote communities can enhance equitable vaccine access. However, the realization of these initiatives hinges on political will and an increase in government health funding allocation. Currently, health funding falls short of the recommended 15% as per the Abuja Declaration (29) which Zimbabwe is a signatory of, and, even when allocated, funds are sometimes inadequately disbursed (30), leaving the healthcare system lacking essential resources to deliver services effectively to the public.

The pandemic underscored the rapid spread of misleading or inaccurate information and the detrimental role that misinformation and disinformation can have in exacerbating vaccine hesitancy. The MOHCC often found itself reacting to myths and misconceptions rather than proactively leading information dissemination. The key lesson here is to prioritize the prompt and widespread sharing of accurate information by the MOHCC as soon as guidelines for a particular disease are released. This approach ensures public awareness and fosters a relationship of trust between the MOHCC and communities, positioning it as a reliable source of verified information.

Investing in STEM education should also be considered more seriously and prioritised in the national budget. While investing in STEM fields may not yield immediate outcomes, the long-term benefits can be substantial, as exemplified by Cuba. Fuelled by relatively generous government support, biomedical researchers in Cuba have managed to excel at creating low-cost vaccines, and developing cancer treatments (31). Such investments empower a nation to cultivate the necessary expertise for conducting research in vaccine development over time.

Additionally, future qualitative research should build upon these findings by explicitly employing established ethical lenses during the analysis of access disparities. Specifically, the Human Rights-Based Approach and principles of distributive justice (e.g., equity, need, and fairness) offer critical frameworks for not only evaluating distribution outcomes but also for guiding policy recommendations designed to mitigate current inequities. Integrating these frameworks is essential for developing a more robust, theoretically-grounded understanding of equitable public health planning.

### Strengths and limitations

The study's main strength lies in its methodology, which leveraged the specialized knowledge and field expertise of individuals directly involved in Zimbabwe's vaccine distribution to gain an in-depth, nuanced understanding of the system. The study also took advantage of online interviews that allowed participants more privacy and anonymity, making them more open to respond to questions. However, it can be argued that a lot of non-verbal communication may have been lost during the interview process as the interviewers and participants were not seeing each other face to face. This limitation is allayed by the inclusion of some face-to face interviews that allowed for diversity on quality of data.

## Conclusion

In conclusion, our study provides valuable qualitative insights into effective strategies for addressing existing vaccine access disparities and promoting sustainable public health measures in Zimbabwe. Our findings highlight several key areas critical for strengthening the national health system:

1. Human Capital and Infrastructure: Key strategies involve investing significantly in STEM education and implementing robust policies to retain healthcare workers, which is essential for strengthening the overall health system.
2. Preparedness and Response: It is necessary to equip health facilities with essential resources and substantially enhance national surveillance and response systems to better anticipate and manage future health crises.
3. Public-Private Partnerships (PPPs): PPPs can and should be utilized as a strategic mechanism to guarantee fair and equitable access to vaccines. This involves coordinating the mobilization and allocation of both financial and human resources to address potential future pandemics effectively.

### Integrating national findings with regional feasibility

By prioritizing these areas, Zimbabwe can bolster the resilience of its health system, fortifying it against future pandemics. Furthermore, the imperative for national self-sufficiency is mirrored at the continental level.

The findings underscore a critical need for regional self-sufficiency to mitigate the catastrophic supply chain disruptions witnessed during the pandemic. Our recommendation for regional vaccine manufacturing partnerships is grounded in concrete policy initiatives already underway across Africa. Specifically, this vision aligns with the Partnerships for African Vaccine Manufacturing (PAVM) initiative (32), spearheaded by the African Union and Africa CDC. The PAVM Framework for Action provides a robust, multi-sectoral roadmap, setting ambitious targets (e.g., meeting 60% of Africa's vaccine demand by 2040) through mechanisms that foster collaboration on R&D, regulatory harmonization, and market design. Such frameworks move the concept of regional self-sufficiency from a conceptual ideal to an actionable, empirically supported policy goal, demonstrating political will and a structured path toward sustainable health security across the continent.

## Data Availability

The original contributions presented in the study are included in the article/Supplementary Material, further inquiries can be directed to the corresponding author/s.

## Footnotes

^1^COVAX information centre | UNICEF.
