# Correction: Equitable access to the COVID-19 vaccines in Africa (ECOVA) findings from a qualitative enquiry in Zimbabwe

**DOI:** 10.3389/frhs.2026.1973708

**Published:** 2026-09-04

**Authors:** Frontiers Production Office

**Affiliations:** Frontiers Media SA, Lausanne, Switzerland

**Keywords:** COVID-19, vaccines, equitable access, vaccine distribution, vaccine access

The funding of the article processing charges was erroneously omitted. The following text has now been added to the Funding statement:

“The article processing charges for this article were funded by the International Development Research Centre (IDRC). The funder had no involvement in the handling, peer review, or editorial decision for this article.”

The original version of this article has been updated.

